# How many SARS-CoV-2 “viroporins” are really ion channels?

**DOI:** 10.1038/s42003-022-03669-2

**Published:** 2022-08-25

**Authors:** Neil L. Harrison, Geoffrey W. Abbott, Martina Gentzsch, Andrei Aleksandrov, Anna Moroni, Gerhard Thiel, Stephen Grant, Colin G. Nichols, Henry A. Lester, Andreas Hartel, Kenneth Shepard, David Cabrera Garcia, Masayuki Yazawa

**Affiliations:** 1 Department of Anesthesiology, Columbia University, New York, NY USA; 2 Department of Physiology and Biophysics, University of California at Irvine, Irvine, CA USA; 3 Marsico Lung Institute, University of North Carolina at Chapel Hill, Chapel Hill, NC USA; 4 Department of Physiology, University of North Carolina at Chapel Hill, Chapel Hill, NC USA; 5 University of Milan, Milan, Italy; 6 Technical University of Darmstadt, Darmstadt, Germany; 7 Yale University, New Haven, CT USA; 8 Washington University School of Medicine, St. Louis, MO USA; 9 Caltech, Pasadena, CA USA; 10 Columbia University, Department of Electrical Engineering, New York, NY USA; 11 Columbia University, Department of Rehabilitation and Regenerative Medicine, New York, NY USA

**Keywords:** Permeation and transport, Viral infection, SARS-CoV-2

**arising from** T. L. Toft-Bertelsen et al. *Communications Biology* 10.1038/s42003-021-02866-9 (2021)

The SARS-CoV-2 virus uses a small number of viral proteins to enter host cells and disrupt their activity, including the spike (S), membrane (M), and envelope (E) proteins, as well as a number of accessory proteins of unknown function (Orf3a, Orf8, Orf10, etc.), some of which may function to alter ion flux across membranes. Toft-Bertelsen et al.^1^ recently reported that the drug amantadine may interact with virally encoded ion channels and proposed that inhibitors of these “viroporins” might have therapeutic use in COVID-19. We concur with the idea that the E protein of SARS-CoV-2 can form an ion channel and that amantadine inhibits this channel, but we suggest below that a number of additional specific criteria need to be met in order for SARS-CoV-2 accessory proteins (Orf3a, Orf8, Orf10) to be accepted as having ion channel activity, and that further work will be necessary. This field represents a neglected area of overlap between biophysics and virology that continues to be understudied and underfunded and clearly merits greater attention from the relevant funding agencies.

Toft-Bertelesen et al.^1^ used *Xenopus* oocytes for their studies, which are phenomenally efficient membrane protein factories and have proven to be a remarkably robust and versatile system for measuring the activity of ion channel proteins and investigating their biophysics and pharmacology. Nevertheless, they do have idiosyncrasies that need to be understood in interpreting the results. The endogenous Ca^2+^-activated channels that are responsible for the fast electrical block to polyspermy in the amphibian egg^2^ are especially relevant to this discussion. This latent conductance dwarfs all others in the uninjected oocyte, and it can be activated by many stimuli that release Ca^2+^ from intracellular stores. The resting membrane potential (RMP) in the oocyte is usually determined by a modest level of activation of Ca^2+^-activated Cl^−^ channels and under these circumstances, the appearance in the plasma membrane of an ion channel permeable to monovalent cations (such as “PM” a plasma membrane-targeted version of the E protein of SARS-CoV-2) results in a sizable (5–10 mV) depolarization of the oocyte membrane (Fig. 1a)^3^, coupled with the appearance of robust membrane currents (Fig. 1b) that are linearly related to the amount of RNA injected (Fig. 1c). In the case of SARS-CoV-2 E protein^1^, these currents are sensitive to amantadine (Fig. 1d, e).

**Fig. 1:**
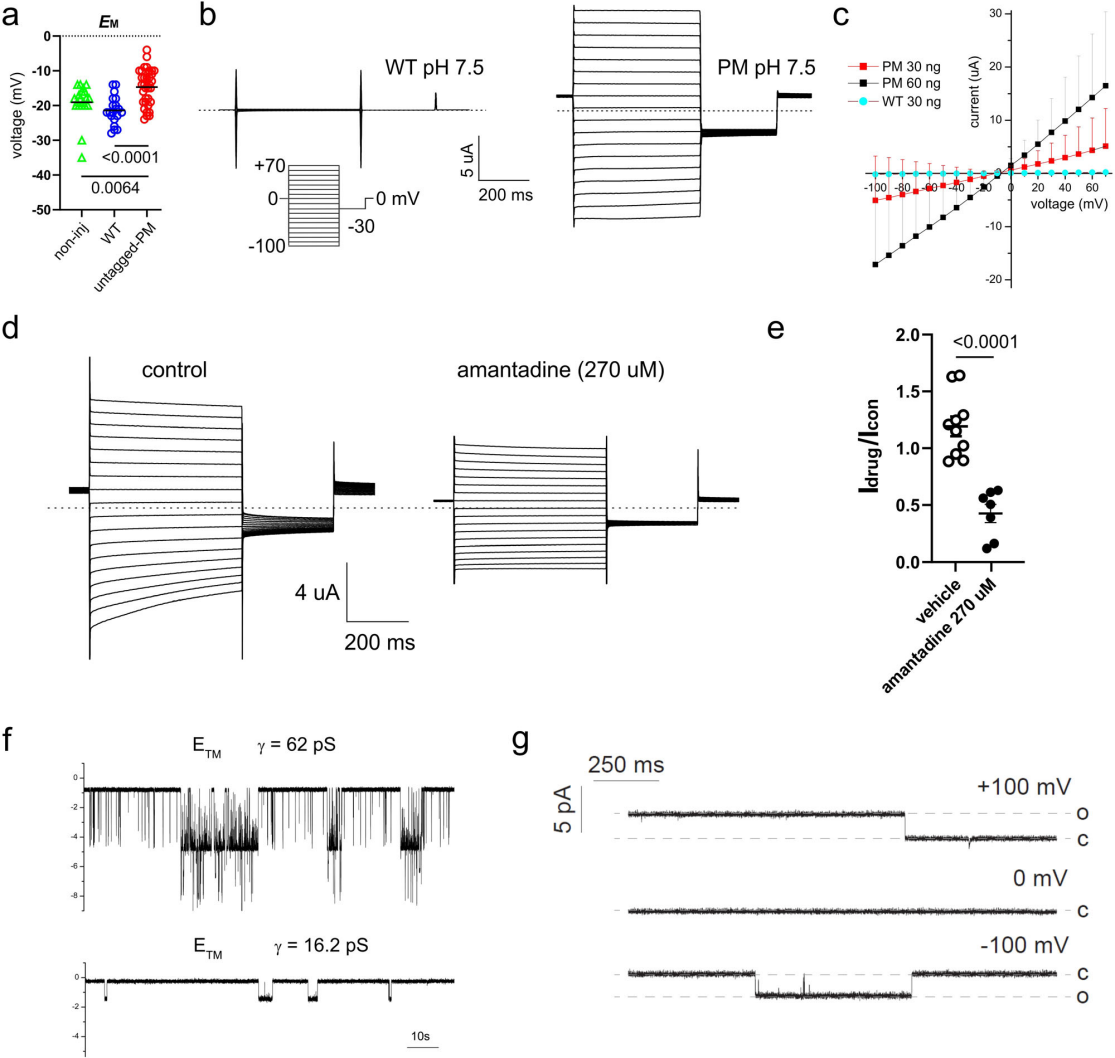
SARS-CoV-2 E protein forms a cation channel in cells and artificial bilayers. **a** Scatter plot of the resting (unclamped) membrane potential (*E*M) for *Xenopus* oocytes expressing untagged-PM (*n* = 35), WT (*n* = 20) or non-injected (non-inj) oocytes (triangles, *n* = 20) (statistical analysis by one-way ANOVA (*P* = 0.0002) and Tukey’s post hoc test, untagged-PM vs. WT, *P* < 0.0001; untagged-PM vs. non-inj, *P* = 0.0064; WT vs. non-inj, *P* = 0.3290). Data reproduced by permission from ref. ^3^. **b** Exemplar current traces for oocytes expressing WT (left panel) and untagged-PM (right panel) as indicated, at pH 7.5 (30 ng cRNA). Voltage protocol and scale bars are shown in the inset. Dashed lines indicate zero current level. Data reproduced by permission from ref. ^3^. **c** Large amplitude (µA) membrane currents on the expression of SARS-CoV-2 E protein are proportional to the quantity of RNA injected. Mean peak current versus voltage for oocytes after injection of 30 ng (red squares, *n* = 17) or 60 ng (black squares, *n* = 8) “untagged-PM” E protein cRNA, or after injection of 30 ng “WT” E protein cRNA (circles, *n* = 15). cRNA encoding WT and untagged-PM constructs was generated from cDNA in the pXOOM vector. Current data shown are mean ± SD, and are reproduced by permission from ref. ^3^. **d** Exemplar current traces for oocytes expressing untagged-PM E protein in the absence (control) or presence of amantadine (270 µM) at pH 7.5 (30 ng cRNA). Voltage protocol and scale bars are shown in the inset. Dashed lines indicate zero current level. Other experimental methods are described in ref. ^3^. **e** Scatter plot of fractional current at −80 mV remaining after incubation of oocytes expressing untagged-PM E protein in bath solution (vehicle) (*n* = 10) or amantadine (270 µM) (*n* = 7) as in (**d**). Statistical analysis performed by unpaired, two-tailed *t* test; bars indicate mean; error bars indicate SEM. **f** Single-channel currents recorded on reconstitution of a synthetic transmembrane (TM) fragment (amino acids 8–38) of E protein into artificial bilayers. The lipoprotein particles used to deliver the TM fragment into the lipid bilayer were prepared by using the protocol described in ref. ^6^ for the pentameric structure formation. Recordings were made in symmetrical 300 mM NaCl, 5 mM MgCl_2_, 10 mM HEPES, pH 7.2, and low-pass filtered at 50 Hz for display purposes. **g** Single-channel currents following the reconstitution of recombinant E protein into artificial bilayers using lipid nanodiscs^5^. E protein from cell-free protein expression in presence of lipid nanodiscs recorded in suspended lipid bilayer 4:1 DPhPC:DPhPS, in symmetrical 250 mM KCl, 10 mM HEPES, pH 7.4, 1 mM EGTA at +100 mV (upper trace), 0 mV (middle trace) and −100 mV (lower trace). Data were low-pass filtered at 500 Hz.

Note also that RNA encoding “WT”, the unmodified wild-type E protein, does not induce the appearance of membrane conductance^3,4^ or depolarize cells in our hands (Fig. 1a–c), but can decrease the appearance of other ion channels in *Xenopus* oocytes, in part by competition for protein-sorting machinery^4^. The wild-type E protein can, however, be reconstituted into artificial bilayers, to allow the detection of well-resolved single-channel currents^5^ (Fig. 1f, g). Depending on experimental conditions, these currents exhibit different unitary conductances of 16 pS and 62 pS, and presumably reflect the assembly of different forms of oligomeric E protein complexes, including pentamers^6^. The above observations are consistent with successful ion channel expression in oocytes and are strikingly reminiscent of what is observed with a classic viroporin: the M2 protein of influenza A virus^7,8^.

In Fig. 4 of Toft-Bertelsen et al., the RMP appears not to be altered by the expression of the SARS-CoV-2 proteins Orf3a and Orf8^1^, although it may be depolarized by expression of Orf10. A straightforward interpretation of the unaltered membrane potential is that RNA injection leads to intracellular trafficking of the E protein, disruption of intracellular homeostasis, Ca^2+^ release from intracellular stores^5^, and activation of the endogenous Ca^2+^-activated channels, leading to a modest conductance increase but no change of RMP. No other viral proteins from SARS-CoV-2 were used that could serve as negative controls^1^. For example, a similar study in oocytes^4^ used the S protein, M protein and Nsp4 protein and showed that none of these proteins gave rise to significant plasma membrane conductances. Taken together, these observations would suggest that caution is appropriate in interpreting the data presented in ref. ^1^ as strongly supportive of ion channel activity of multiple proposed “viroporins” for SARS-CoV-2.

In our collective recent studies of the E protein of SARS-CoV-2, we were aware of the many potential interpretational problems and several members of this group used fluorescent tags to assist in elucidating localization of the protein to intracellular membranes^3,5^. We all agree that the primary location of the wild-type E protein in cells is within intracellular organelles^3–5^, at the endoplasmic reticulum–Golgi intermediate compartment (ER-GIC, refs. ^3,5^) and associated structures, where it may act to promote viral assembly via the deacidification of this cellular compartment^3,5^. We suspect that other viral accessory proteins may also be localized to this or neighboring organelles prior to virion assembly and release. Certainly, this seems to be the case for the N, M, S, and E proteins following the infection of Vero cells by SARS-CoV-2^9^ and this subcellular compartmentalization would be expected to limit the ability to study the possible ion channel activity of these and other SARS-CoV-2 proteins.

In our experiments, the strong ER retention signal of SARS-CoV-2 E was removed and replaced with an export signal (PM; Fig. 1b, c), leading to the efficient expression of the E protein in the plasma membrane of HEK 293 cells and oocytes^3^, generating membrane currents that were not seen following expression of the WT protein^3–5^. We proposed the initial minimal criterion that only those viral proteins that produce robust membrane conductances can be considered to generate ion channels^8^, and we wanted to test this idea quantitatively. To define and quantify what constitutes a “robust” conductance in oocytes, we know that a typical oocyte has a capacitance of 200 nF^4^. At 0.01 pF/μm^2^, we therefore estimate that the oocyte has ~2 × 10^7^ μm^2^ of membrane. Leak (voltage-independent) current in healthy oocytes is usually considered to be of the order of 100–200 nA at −100 mV, so calcium-activated chloride currents are associated with a leak conductance ~200 nA/100 mV = 2 µS, or about 1 pS/μm^2^. Healthy oocytes used in ion channel experiments are typically employed to measure currents >1 µA, so 1 µA/100 mV = 10 µS, yielding a conductance of 5 pS/μm^2^ of oocyte membrane. Using the results of ref. ^5^ to provide a value for the E protein single-channel conductance (16 pS), we can therefore estimate that Cabrera et al.^3^ expressed >0.3 channels/μm^2^; by most physiological criteria, this is a rather low level of channel protein density.

If we propose that a minimum value of 5 pS/μm^2^ satisfies the requirement for generating a robust RNA-induced plasma membrane conductance in oocytes, then this represents a minimum signal-to-noise ratio of 5 for the E protein current over the endogenous background current. In the report of ref. ^1^, the data suggest that the apparent E protein-dependent current density was close to our proposed threshold criterion, while the Orf3a-dependent current density was only half as large^1^. At the time of writing, only the E protein of SARS-CoV-2 satisfies our basic criterion, and then more obviously when it is re-engineered to appear at the cell membrane in *Xenopus* oocytes^3^. Furthermore, the E protein of SARS-CoV-2 produces single-channel conductance transitions when reconstituted into artificial bilayers (ref. ^5^, Fig. 1f, g). These findings are collectively similar to those observed with other established viroporins that have been studied in the past, such as the M2 protein of influenza virus^7^.

In view of all the above observations, we propose that the following minimal criteria be met in order for a proposed viroporin^8^ to be considered as generating an ion channel^8^: viral protein must be shown to (a) produce robust membrane conductance when expressed at the plasma membrane^3^, (b) be associated with characteristic single-channel currents, (c) show specific pharmacology^1^ (d) display ion selectivit*y*^3^, and (e) show mutations that alter ion channel function. The E protein of SARS-CoV-2 already satisfies most of these criteria^1,3^, while much additional work needs to be done in this area to verify and define the possible ion channel activity of other suspected viroporins.

## Data Availability

All data are available from the corresponding authors upon request.
